# Supplementary figures and images for: Occurrence and abundance of microplastics in surface water of Songkhla Lagoon
Source: PeerJ. 2024 Jul 26;12:e17822. doi: 10.7717/peerj.17822 (PMC11285385; doi:10.7717/peerj.17822)

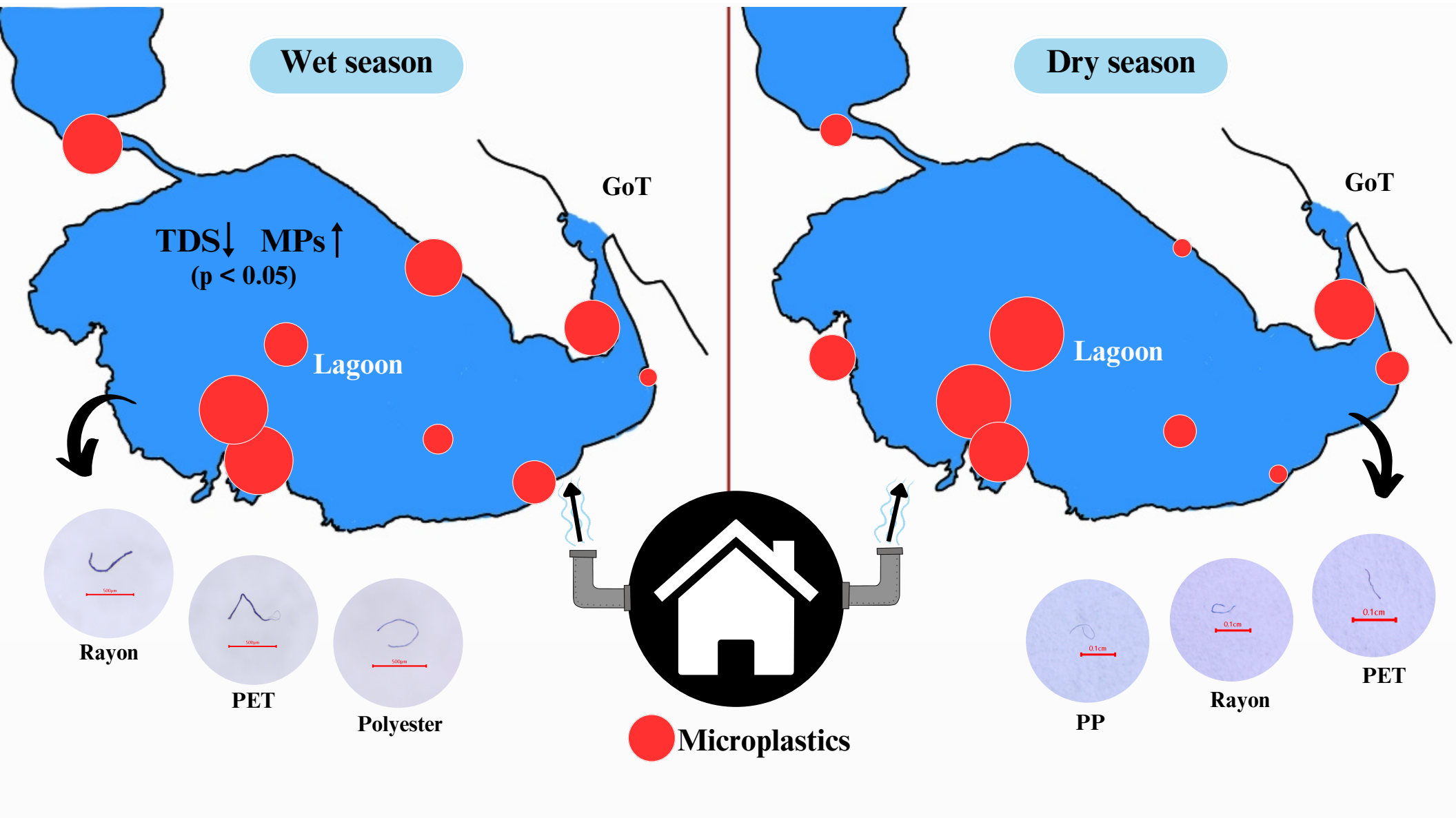

Supplement: Supplemental Information 1 — Source credit: GEO-Informatics Center for Natural Resources and Environment, Prince of Songkla University, Thailand. [file peerj-12-17822-s001.pdf]
